# Utilizing *FMR1* Gene Mutations as Predictors of Treatment Success in Human In Vitro Fertilization

**DOI:** 10.1371/journal.pone.0102274

**Published:** 2014-07-14

**Authors:** Vitaly A. Kushnir, Yao Yu, David H. Barad, Andrea Weghofer, Eric Himaya, Ho-Joon Lee, Yan-Guang Wu, Aya Shohat-Tal, Emanuela Lazzaroni-Tealdi, Norbert Gleicher

**Affiliations:** 1 Center for Human Reproduction, New York, New York, United States of America; 2 Foundation for Reproductive Medicine, New York, New York, United States of America; 3 Department of Obstetrics and Gynecology, University of Vienna School of Medicine, Vienna, Austria; 4 Gatineau Hospital, McGill University, Quebec, Canada; Michigan State University, United States of America

## Abstract

**Context:**

Mutations of the *fragile X mental retardation 1* (*FMR1*) gene are associated with distinct ovarian aging patterns.

**Objective:**

To confirm in human in vitro fertilization (IVF) that *FMR1* affects outcomes, and to determine whether this reflects differences in ovarian aging between *FMR1* mutations, egg/embryo quality or an effect on implantation.

**Design, Setting, Patients:**

IVF outcomes were investigated in a private infertility center in reference to patients' *FMR1* mutations based on a normal range of CGG_n = 26–34_ and sub-genotypes *high* (CGG_n>34_) and *low* (CGG_<26_). The study included 3 distinct sections and study populations: (**i**) A generalized mixed-effects model of morphology (777 embryos, 168 IVF cycles, 125 infertile women at all ages) investigated whether embryo quality is associated with *FMR1*; (**ii**) 1041 embryos in 149 IVF cycles in presumed fertile women assessed whether the *FMR1* gene is associated with aneuploidy; (**iii**) 352 infertile patients (< age 38; in 1^st^ IVF cycles) and 179 donor-recipient cycles, assessed whether the *FMR1* gene affects IVF pregnancy chances via oocyte/embryo quality or non-oocyte maternal factors.

**Interventions:**

Standardized IVF protocols.

**Main Outcome Measures:**

Morphologic embryo quality, ploidy and pregnancy rates.

**Results:**

(**i**) Embryo morphology was reduced in presence of a *low FMR1* allele (P = 0.032). In absence of a *low* allele, the odds ratio (OR) of chance of good (vs. fair/poor) embryos was 1.637. (**ii**) *FMR1* was not associated with aneuploidy, though aneuploidy increased with female age. (**iii**) Recipient pregnancy rates were neither associated with donor age or donor *FMR1*. In absence of a *low FMR1* allele, OR of clinical pregnancy (vs. chemical or no pregnancy) was 2.244 in middle-aged infertility patients.

**Conclusions:**

A *low FMR1* allele (CGG_<26_) is associated with significantly poorer morphologic embryo quality and pregnancy chance. As women age, *low FMR1* alleles affect IVF pregnancy chances by reducing egg/embryo quality by mechanisms other than embryo aneuploidy.

## Background

Though currently still considered a gene with primarily adverse neuro-psychiatric associations, the *fragile X mental retardation 1* (*FMR1*) gene, located at Xq27.3 [1], has in recent years also attracted attention because of its apparent involvement in regulating ovarian aging [2]–[5]. A recent publication conclusively demonstrated in the human differences in decline of functional ovarian reserve (FOR) with different *FMR1* mutations (genotypes and sub-genotypes) [6].

These mutations are based on a newly described normal range, defined by CGG_n = 26–34_. This range allows for the definition of mutations based on CGG_n_ on both alleles of the X chromosome, described as genotypes/sub-genotypes [2], [3], [7], [8]. Aside from demonstrating *FMR1* mutation-specific declines in FOR [2], [3], [7], [8], different *FMR1* mutations also have been associated with varying pregnancy chances in association with in vitro fertilization (IVF) [7], [8], an observation suggesting that, as women age, the *FMR1* gene may not only affect FOR, but also chance of conception.

Since *FMR1* mutations also appear associated with autoimmune-risk [7], [8], and implantation is an immunologic process [9], how the *FMR1* gene affects pregnancy chances (via oocyte/embryo quality or other non-oocyte maternal factors) is, therefore, of interest.

This study investigated this question, utilizing distinct patient populations of young oocyte donors, middle-aged women of presumed normal fertility and infertile middle-aged women in treatment. The latter patient population included a subset of women who used donor eggs, allowing for assessment of possible donor *FMR1* effects mediated by oocyte quality alone and allowing comparison to those who were using their own oocytes.

## Methods

### Study Participants

This study investigated three distinct patient populations: (i) 777 embryos in 168 IVF cycles in 125 infertile women of all ages, assessing whether embryo quality differs in association with *FMR1* mutations. (ii) In assessing whether the *FMR1* gene is associated with risk of aneuploidy, 1041 embryos in 149 IVF cycles in presumed fertile women undergoing IVF for pre-implantation genetic diagnosis; and (iii) in a third model 179 consecutive oocyte recipient IVF cycles, in determining whether mutations of the *FMR1* gene in donors affect IVF pregnancy chances in recipients. This third model in addition utilized as comparison group 352 consecutive infertile patients (mean age 33.4±3.4 years), who used their own eggs in first IVF cycles.

Since *low* (CGG _n<26_) *FMR1* alleles have previously been associated with significant declines in IVF pregnancy rates [7], [8], two questions were posed by this model: first, whether with *low FMR1* alleles a similar decline in pregnancy chances can be confirmed as previously reported, this time in another infertile patient population; and, second, whether a decline in pregnancy chance would also be observed in an egg donation model, where older patients receive oocytes from young oocytes donors with different *FMR1* mutations.

All patients and oocyte donors sign informed consents at time of initial consultation, allowing the use of their medical record for research purposes, as long as their identity is protected and the medical record remains confidential. Both conditions were met for this study, qualifying it for expedited review by the center's institutional review board (IRB).

Our center accepts less than five percent of oocyte donor applicants. Donor selection involves an initial screening step by questionnaire, followed by two rounds of face-to-face interviews and a medical testing round. Screening excludes donor candidates with presumed increased reproductive risks, based on their medical, family and genetic histories.

Full mutation (CGG_n>200_) and premutation range alleles (CGG_n = 55–200_), long known associated with POI/POF, were absent in investigated populations. Here presented *FMR1* data on *high* alleles (CGG_n>34_), therefore reflect on women with what currently are considered normal CGG _n<45_ or “gray-zone” CGG _n∼45–54_ CGG repeats, and cannot be extrapolated to women with premutation or full mutation range alleles.

### Art Protocols

Patients and donors underwent standardized ovarian stimulation protocols as previously described [6], [8]. Briefly, patients under age 40 with normal functional ovarian reserve (FOR) received down regulation with full dose (1.0 mg/0.1 mL) gonadotropin releasing hormone agonist (GnRH-a; Lupron, Abbot Pharmaceuticals, North Chicago, IL) and ovarian stimulation with up to 300 IU of gonadotropins daily, usually half as follicle stimulation hormone (FSH) and half as human menopausal gonadotropins (hMG).

Patients with diminished FOR and/or low serum androgens and those over age 40 received at least six weeks of dehydroepiandrosterone (DHEA) supplementation with 25 mg t.i.d. of pharmaceutical grade, micronized DHEA prior to IVF cycle start, as previously described [10]. Their cycles involved prevention of premature ovulation with microdose GnRH-a (50 µg/0.1 mL, b.i.d.), and ovarian stimulation with 300–450 IU FSH and 150 IU of hMG daily.

Oocyte donors underwent down regulation with full dose GnRH-a (1.0 mg/0.1 mL) and ovarian stimulation with up to 300 IU of hMG daily.

Final oocyte maturation was triggered in all cycles with 5,000–10,000 IU of human chorionic gonadotropin (hCG).

### Laboratory Assesments

#### *FMR1* Testing

Assessments of CGG_n_ of the *FMR1* gene was performed by commercial assays, as previously described, with *FMR1* mutations (genotypes and sub-genotypes) defined as described in prior publications [2], [3], [7], [8]. In brief, by defining a normal CGG _n = 26–34_ range, CGG counts below and above that range are abnormal. A female with both *FMR1* alleles in normal range, therefore, is *norm*, one with one in and one outside normal range is *het* and one with both alleles outside norm range is *hom*. Whether an allele is above (*high*) or below (*low*) normal range further sub-divides *het* and *hom* genotypes into sub-genotypes (*het-norm/high*, *het-norm/low*; *hom-high/high*, *hom-high/low*, *hom-low/low*).

In this study, included oocyte donors and infertility patients had *FMR1* testing performed to exclude FXS risks in offspring. Based on the center's IRB instructions, the center during the study period did not consider *FMR1* mutations in either selecting egg donors and/or in selecting treatments of infertile patients.

#### Determination of Embryo Morphology

Cleavage stage embryos were classified as good (4 cells d-2, 8 cells d-3, little or no fragments), poor (arrested embryos or >25% fragmented) or fair (all other embryos).

#### Determination of Embryo Ploidy

Preimplantation genetic screening (PGS) was performed in a group of 121 fertile women undergoing a total of 149 IVF cycles for non-infertility related reasons, primarily elective gender selection. Embryos were biopsied on day three after fertilization at 6–8 cell stages. Fluorescence in situ hybridization (FISH) was utilized with probes for seven chromosomes (X, Y, 13, 16, 18, 21 and 22). Reported aneuploidy rates are, therefore, incomplete but should not influence *FMR1* mutation-associated findings.

### Statistical Analysis

This study investigated the relationship between *FMR1* mutations (genotypes/sub-genotypes) and embryo morphology, embryo ploidy and clinical pregnancy rates, while accounting for the variability of age. Since presence of *low* alleles (CGG_n<26_) in prior studies impacted FOR as well as pregnancy chance with fertility treatment [8], analyses in this study primarily compared patients with *low* alleles to those without *low* alleles.

Generalized mixed-effects (GLME) models were applied to examine embryo morphology and ploidy based on *FMR1* genotype. Generalized estimating equation (GEE) models were utilized to confirm GLME results. A logistic regression model was used to study the clinical pregnancy rate of first IVF treatments in infertility patients under age 38.

All statistical analyses were adjusted for female age. The analysis of embryo morphology was also adjusted for number of prior treatment cycles. Covariates were considered statistically significant when P values were <0.05 using SAS 9.2. The center's senior statistician (Y.Y.) performed all analyses.

## Results

### (i) *Fmr1* And Morphological Embryo Quality

We here investigated 777 embryos from 125 women in 168 IVF cycles in infertile women of all ages. Table 1 summarizes patient characteristics: Mean age of this patient population was 39.7±5.7 years. This infertile patient group, therefore, was not restricted in age. A little less than half (45.4%) of all embryos were considered of good quality, 43.4% fair and 11.2% of poor quality.

**Table 1:** Characteristics of infertile patients in section (i).

Variables	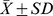 or *n (%)*		
Number of patients	125		
Number of cycles	168		
Age (years)	39.7±5.7		
Race (African; Asian; Caucasian; other)	9.7%; 15.3%; 74.2%; 0.8%		
FSH (mIU/mL)	11.2±12.5		
AMH (ng/mL)	1.5±1.9		
Number of embryos	777		
Embryo quality (Good/Fair/Poor)	353(45.4%)/337(43.4%)/87(11.2%)		
***FMR1*** ** mutations**	**Patients ** ***n(%)***	**Cycles ** ***n(%)***	**Embryos ** ***n%)***
* Norm*	51(40.8%)	60(35.7%)	319(41.1%)
* het-norm/high*	25(20.0%)	34(20.2%)	144(18.5%)
* hom-high/high*	1(0.8%)	1(0.6%)	3(0.4%)
* het-norm/low*	37(29.6%)	56(33.3%)	234(30.1%)
* hom-low/low*	4(3.2%)	6(3.6%)	41(5.3%)
* hom-low/high*	7(5.6%)	11(6.6%)	36(4.6%)

Women with *low* sub-genotypes (CGG_n<26_) were overrepresented and those with *high* sub-genotype (CGG_n>34_) and *norm* genotypes underrepresented in comparison to prior reports [5], [7], [8], [11]. Here investigated infertile women, therefore, are likely more adversely selected than previously reported infertile populations.

**Figure 1:**
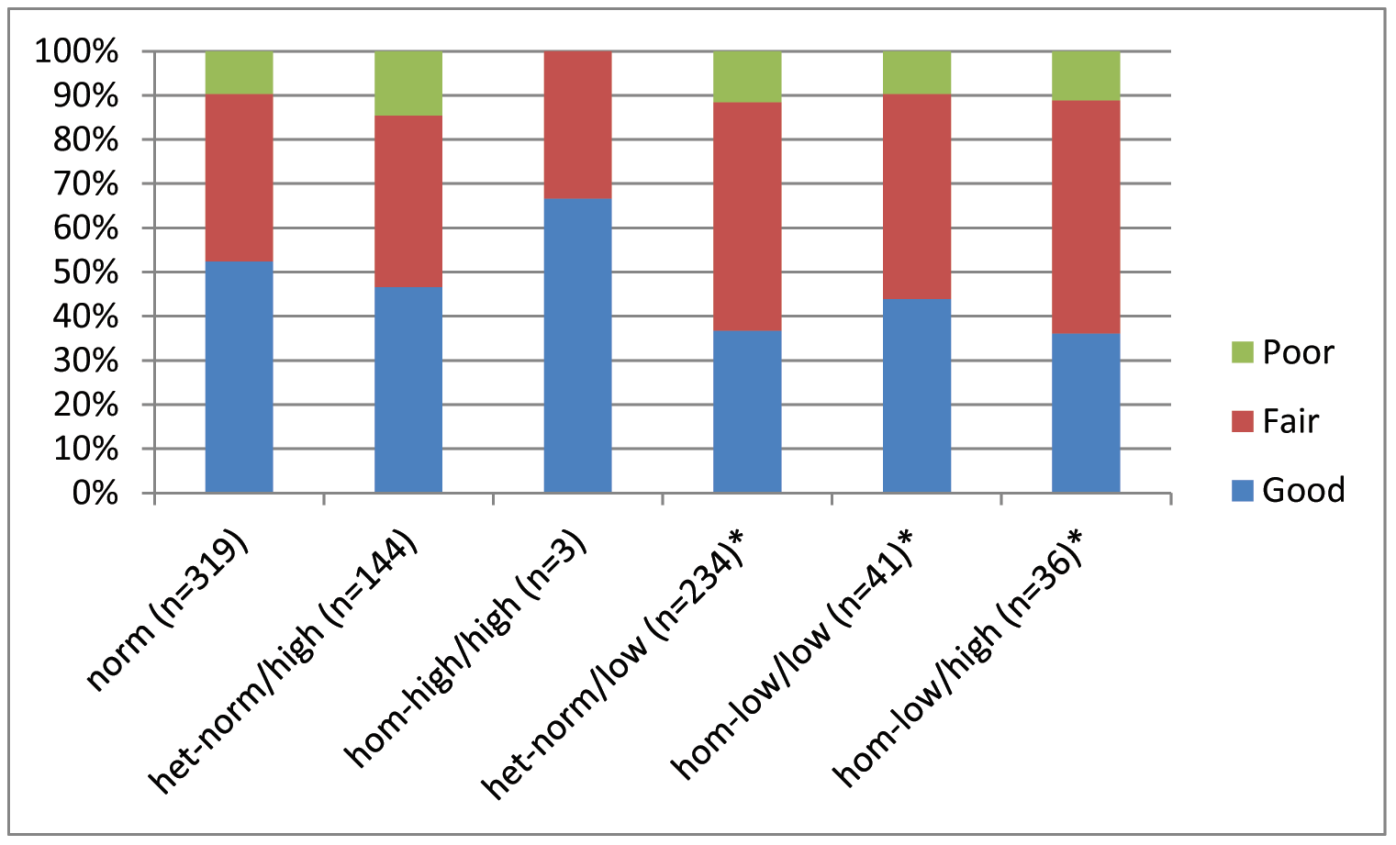
The distribution of morphologic embryo quality for each *FMR1* sub-genotype. *Morphologic embryo quality in sub-genotypes with a *low* allele present was significantly reduced in comparison to those without a *low* allele (P = 0.032). OR of chance of good vs. fair/poor embryos in absence of a *low* allele was 1.637.

Figure 1 summarizes morphologic embryo quality, based on *FMR1* genotypes/sub-genotypes. Comparing availability of good quality embryos to availability of fair and poor quality embryos, morphologic embryo quality in women with at least one *low FMR1* allele was statistically different from patients with only *norm* (CGG_n = 26–34_) and *high* (CGG_n>34_) alleles (P = 0.03). Odds ratio (OR) estimate of having good morphologic quality embryos vs. having fair and/or poor quality embryos between *low* and *norm* and/or *high* genotypes/sub-genotypes was 1.637, indicating that patients with only *norm* and/or *high* alleles had a 63.7% higher probability of producing good morphologic quality embryos than patients with at least one *low FMR1* allele.

### (ii) *Fmr1* And Embryo Aneuploidy Rates

Since embryo ploidy represents a substantial component of total functional embryo quality [12], embryo ploidy was assessed next. This assessment was made in 1041 embryos from 149 IVF cycles in presumably fertile women undergoing IVF with preimplantation genetic diagnosis (PGD) for non-infertility related reasons, mostly elective gender determination. This patient group represented a mid-range age (33.5±5.5 years), and was, therefore, somewhat younger than in section (i) investigated infertility patients. Table 2 summarizes patient characteristics.

**Table 2:** Characteristics in section (ii) of women undergoing IVF for non-fertility related indications.

Variables	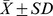 or *n(%)*		
Patients	121		
Number of cycles	149		
Age (years)	33.5±5.5		
Race (African; Asian; Caucasian; other)	11%; 23%; 65%; 1%		
FSH (mIU/mL)	8.7±3.1		
AMH (ng/mL)	3.2±2.6		
Number of embryos	1041		
Ploidy (normal/abnormal)	571(54.9%)/470(45.2%)		
***FMR1 sub-genotypes***	**Oocyte source ** ***n(%)***	**Cycles ** ***n(%)***	**Embryos ** ***n(%)***
*norm*	71(58.7%)	85(57.1%)	646(62.1%)
*het-norm/high*	19(15.7%)	23(15.4%)	141(13.5%)
*hom-high/high*	2(1.7%)	3(2.0%)	26(2.5%)
*het-norm/low*	26(21.5%)	34(22.9%)	193(18.5%)
*hom-low/low*	2(1.7%)	2(1.3%)	16(1.5%)
*hom-low/high*	1(0.8%)	2(1.3%)	19(1.8%)

As the table demonstrates, the *FMR1* mutation distribution in this presumed fertile female population differed from in section (i) investigated infertile patients (P<0.001), and is closer to previously reported distribution patterns, demonstrating predominantly more *norm* genotypes and fewer *het* as well as *hom FMR1* mutations with *low* as well as *high* alleles. Most remarkable is, however, the remarkably lower rate of *low* alleles, suggesting a possible association between *low* alleles and infertility in older women.

**Figure 2:**
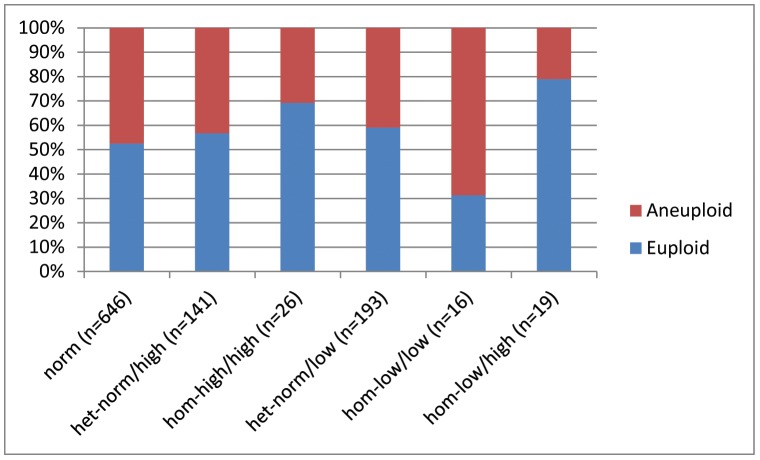
The distribution of embryo ploidy based on patients *FMR1* sub-genotype. *FMR1* mutations were not statistically associated with embryo ploidy, though the high aneuploidy rate in biallelic *low*, *hom-low/low* women is quite remarkable. Lack of significance may in here-reported findings, therefore, be consequence of small patient numbers. Also of interest is the very low aneuploidy rate in women with one *low* and one *high* allele, presenting with only approximately half the aneuploidy rate of even *norm* women. While these findings, dues to small patient numbers, also need to be viewed with caution, they could suggest a compensatory benefit from a *high* allele on negative effects of a *low* allele.

Figure 2 summarizes aneuploidy rates in reference to *FMR1* mutations. No statistical differences in aneuploidy rate were noted between women with *low* and/or *norm* and *high* alleles (OR, 0.855; 95% CI 0.578, 1.266; P = 0.434). Interestingly, biallelic *low* women, however, did demonstrate unusually high aneuploidy rates, suggesting that lack of significant findings in this section of the study could be due to relatively small study subject numbers. Also of interest is the very low aneuploidy number in women with one *low* and one *high* allele, suggesting a potential compensatory effect of a *high* allele on potential negative effects of a *low* allele. Since both of the latter observation occurred in only small patient subsets, and did not reach statistical significance, they should, however, as of this point only be considered hypothesis generating.

As would be expected, age of women yielding oocytes was statistically related to aneuploidy (OR 1.041; 95%CI 1.011, 1.072; P = 0.007): one-year increase of advancing female age resulted in a 4.1% higher chance of embryo aneuploidy.

These results suggest that the relationship demonstrated in section (i) between morphologic embryo quality and *FMR1* mutations is not, likely, based on embryo ploidy.

### (iii) Effect Of *Fmr1* On Clinical Pregnancy Rates

In infertile women *FMR1* mutations (genotypes/sub-genotypes) have previously been demonstrated predictive of IVF pregnancy chances [7], [8]. Since IVF pregnancy chances are known associated with embryo quality [13] and, since results in section (i) demonstrate that *norm* and/or *high FMR1* alleles are associated with significantly more good quality embryos than *low* alleles, one can conclude that the *FMR1* gene affects IVF pregnancy chances via oocyte/embryo quality.

IVF pregnancy chances can, however, also be significantly affected by implantation, considered an immunologically-influenced process [9], [14]. *Low FMR1* alleles in infertile women have also been associated with abnormal immune laboratory findings in infertile women, suggestive of immune system activation [7], [8]. Adverse effects of the *FMR1* gene on implantation, therefore, cannot be ruled out.

Section (iii) of this study was, therefore, designed to differentiate between egg/embryo quality or implantation effects as cause for lowered IVF pregnancy rates in association with *low FMR1* alleles. It represents a study, investigating 352 first autologous IVF cycles in middle-aged infertile women under age 38 (mean 33.4±3.4 years) and 179 donor/recipient IVF cycles, utilizing donated eggs from 162 young oocyte donors (mean donor age 25.0±2.9 years). As expected, the clinical pregnancy rate was higher in donor (55.9%) than autologous IVF cycles (28.4%; P<0.001).

Due to the small sample sizes of *hom-low/low, hom-high/high and hom-low/high* women, we in this study section combined all *hom* patients into one group when comparing the distribution of *FMR1* mutations between the two sub-study groups in this section.

Distribution of *FMR1* genotypes/sub-genotypes significantly differed between the two study groups in this section (P<0.001). Interestingly, the here investigated younger infertile patient group demonstrated a similar distribution of *FMR1* mutations to the presumed fertile women in section (ii), while young oocyte donors presented with a distribution in-between these two middle-aged groups and in section (i) investigated much older infertile patients (Table 3). The distribution pattern seen in oocyte donors, therefore, likely is typical for young normal female populations, suggesting in young women an approximately 22% prevalence of *low FMR1* alleles but a much higher prevalence in older infertile women. Once again, the increasing prevalence of *low* alleles, comparing young donors and older infertile women points towards a potential association of *low FMR1* alleles with infertility at advanced ages.

**Table 3:** Donor egg recipients and infertility patients using autologous oocytes included in section (iii).

	_Donor/Recipients_	_Infertile patients_
Variables	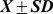 or *n(%)*	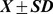 or *n(%)*
Donors	127	-
Recipients and Infertile women	162	352
Cycles	179	352
Age – Oocyte Source (years)	25.0±2.9	33.4±3.4
Race – Oocyte Source (African; Asian; Caucasian; other)	7.3%; 12.9%; 75.3%; 4.5%	11.9%; 15.2%; 70.0%; 2.9%
AMH – Oocyte Source (ng/mL)	4.0±2.3	1.9±2.0
Clinical pregnancy rate	100(55.9%)	100(28.4%)
***FMR1*** ** sub-genotypes**	***n*** **(%)**	***n*** **(%)**
(of women reaching retrieval)		
*norm*	90(50.3%)	217(61.7%)
*het-norm/low*	51(28.5%)	63(17.9%)
*het-norm/high*	17(9.5%)	55(15.6%)
*hom-low/low*	5(2.8%)	10(2.8%)
*hom-high/high*	9(5.0%)	3(0.9%)
*hom-low/high*	7(3.9%)	4(1.14%)

**Figure 3:**
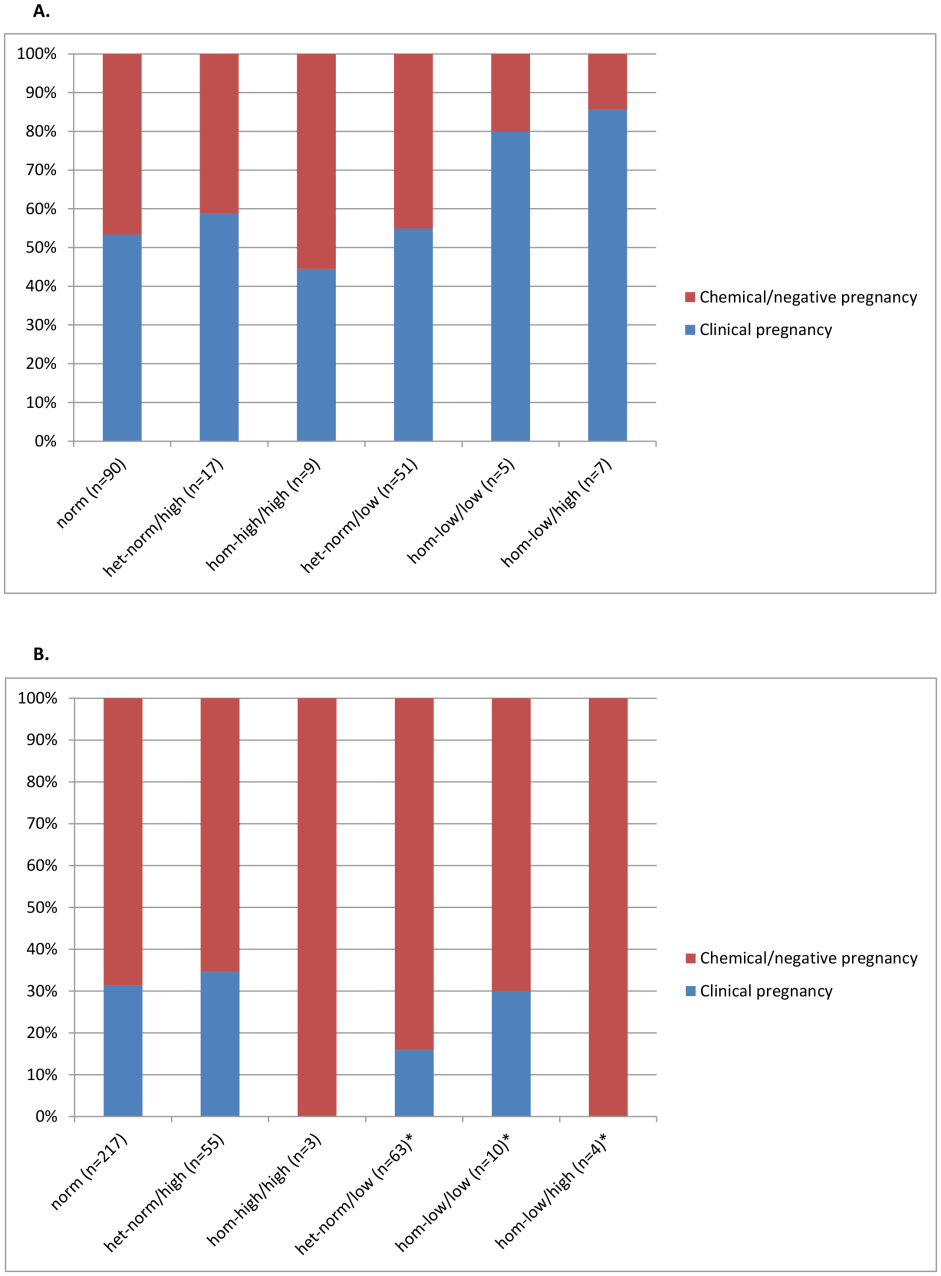
The distribution of clinical pregnancies based on *FMR1* sub-genotype of Clinical pregnancy rates in A oocyte donors and B middle-aged infertility patients. Oocyte recipient pregnancy rates were not associated with donor *FMR1*. Using a logistic regression model in middle-aged infertility patients, OR of clinical pregnancy vs. chemical or no pregnancy was 2.244 in absence of a *low FMR1* allele vs. presence of *low* alleles (P = 0.0015), suggesting 1.244-times odds of clinical IVF pregnancy in absence of a *low FMR1* allele.

Figures 3A and 3B demonstrate clinical pregnancy rates in association with *FMR1* mutations. Once again comparing women with at least one *low FMR1* allele to those with only *norm* and *high* alleles, Figure 3A demonstrates that in donor-recipient cycles the *FMR1* mutation of the donor did not affect recipient pregnancy rates (OR 0.738; 95% CI 0.387, 1,405; P = 0.347). Moreover, donor ages also did not affect pregnancy chances (OR 0.970; 95%CI 0.874, 1.078; P = 0.568).

Using a logistic regression model, in young infertility patients under age 38 years, odds of clinical pregnancy, however did differ significantly between patients with single *low FMR1* alleles and those with only *norm* and/or *high* alleles (OR 2.244; 95%CI 1.168, 4.312; P = 0.015; Figure 3B). Odds of clinical pregnancy vs. biochemical or no pregnancy between both groups were 2.244. The odds ratio estimate, thus, indicates that women with only *norm* and/or *high FMR1* alleles have 1.244-times higher probability of clinical pregnancy than women with *low* alleles, and confirming our earlier reports [7], [8].

**Figure 4:**
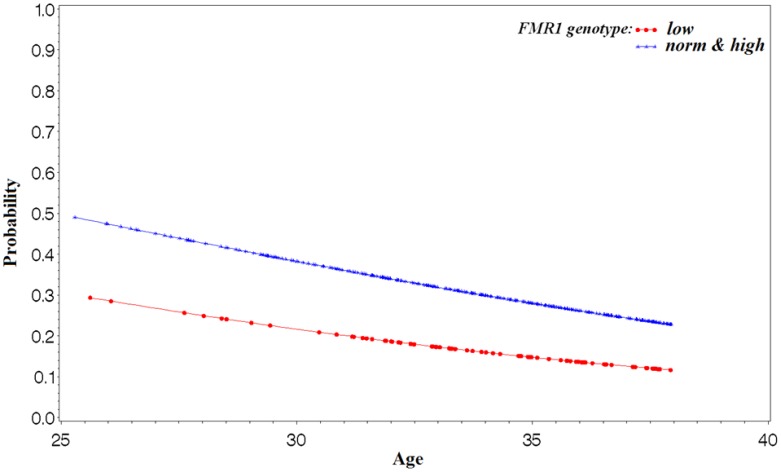
Predicted probabilities of clinical IVF pregnancy in infertile patients based on age and *FMR1*.

Figure 4 demonstrates that the difference in odds of clinical pregnancy in this relatively young group of infertile women remains remarkably stable with advancing female age between women with *low* and with *norm/high* alleles, though it does minimally narrow as women age.

Prior IVF cycle numbers (at other centers) and patient age were statistically not related to morphologic embryo quality (data not shown).

## Discussion

By demonstrating that specific *FMR1* gene mutations are associated with morphologic embryo quality and with chance of clinical pregnancy in association with IVF, this study establishes the *FMR1* gene as the first gene statistically associated with IVF outcomes. This also means that the gene is not only, as previously reported, associated with ovarian aging by affecting FOR [2], [3], [5], [7], [8], but also affects embryo quality. Since embryo quality is largely dependent on egg quality [15], it, as of this point, remains to be determined whether the *FMR1* gene impacts the oocyte or, directly, the embryo. The study also demonstrates that this genetic effect persists at all ages (Figure 4).

Interestingly, while embryo ploidy is generally considered to represent a large part of functional embryo competence, this study suggests that the morphologic differences in embryo quality between *FMR1* mutations were, likely, not ploidy-related. These observations may at least partially explain why embryo morphology is only relatively poorly associated with embryo ploidy [16], and why the procedure of PGS so far has failed to improve IVF outcomes [17].

Here reported ploidy-related finding should, however, be viewed with some caution since they may not be applicable to all infertility patients undergoing IVF: Section (ii) of the study was performed in presumably fertile women, most undergoing IVF for non-fertility relates issues. Our center routinely supplements women with low age-specific FOR with DHEA [10] in order to raise androgen levels, in such patients reported to be low [18]. DHEA supplementation, in turn, appears to lower aneuploidy rates in patients with low FOR [19]. Treatment with DHEA in some patients in this study group may, therefore, have affected the results of here reported ploidy investigation.

Significant differences in *FMR1* mutation distribution between the different study groups investigated in the three sections of this study were an unexpected finding. In previous studies infertile patients demonstrated in slightly more than half of cases *norm* genotypes, in slightly more than 40% *het* sub-genotype, *het-low* slightly exceeding *het-high*, and in under 10% the three *hom* sub-genotypes [2], [3], [5], [7], [8].

In this study infertility patients of very advanced age (mean 39.7±5.7 year) in section (i) deviated from this distribution most, demonstrating few *norm* genotypes (41.1%) and a very high prevalence of *low* alleles. In contrast, presumed fertile middle-aged patients in section (ii) (mean age of 33.5±5.5) presented with high numbers of *norm* genotypes (62.1%) and relatively low numbers of monoalleleic *low* patients. Middle-aged infertility patients in section (iii) of the study also presented with a high number of *norm* genotypes (61.7%) and a relative low number of monoalleleic *low* patients.

Interestingly, young and presumed healthy oocyte donors (mean age 25.0±2.9 years) presented with exactly half (50.3%) *norm* genotypes, 28.5% *het-norm/low* sub-genotypes and only 9.5% *het-norm/high* sub-genotypes. Overall, 35.2% of the center's oocyte donors undergoing egg retrieval carried a monoalleleic *low FMR1* gene.

Biallelic *low* carriers even at young ages already demonstrate abnormally low FOR, and *low* monoalleleic carriers, already at young ages lose FOR more rapidly than other *FMR1* mutations [6]. This study now demonstrates that monoalleleic *low FMR1* alleles are also associated with poor embryo quality; yet despite approximately one-third of all here investigated oocytes donors carrying a *low FMR1* allele, donor/recipient pregnancy rates were not affected by a donor's *low* allele. The clinical pregnancy rate was, indeed, higher in donors carrying a *low FMR1* allele (60.3%) than donors with *norm* and/or *high FMR1* alleles (53.5%), though this difference did not reach statistical significance (P = 0.3767).

On first impression contradictory, these results actually confirm prior publications. We [20] and others [21] previously demonstrated how difficult it is to detect adverse *FMR1* effects in young egg donors. Here presented data confirm this fact and again suggest that at such young ages enough ovarian function redundancy may exists to obviate existing defects.

An alternative explanation would be that, as noted earlier, *FMR1* effects occur only later in life and, therefore, are not yet apparent in young oocyte donors. Such an explanation, however, appears less likely since *FMR1* related differences in IVF pregnancy rates are already apparent at relative young ages, and differences between monoalleleic *low* and all other *FMR1* mutation carriers do not change dramatically with age (Figure 4).

Moreover, at least in rodents, fragile X mental retardation protein (FXMRP) and *FMR1* mRNA appear already expressed during all stages of follicle development [22], thus suggesting a possible direct *FMR1* effect on oocytes.

While at young ages redundancy of ovarian reserve, likely, among those with *low FRM1* alleles accounts for no observed decrease in pregnancy rates in donor cycles, redundancy does not necessarily also protect cumulative pregnancy chances over sequential IVF cycles, utilizing fresh and frozen embryos. Unless egg donors are very young, exclusion of those with *low FMR1* alleles and/or low ovarian reserve may, therefore, be appropriate to optimize cumulative pregnancy rates based on here presented and recently reported data [6].

Many IVF programs currently test oocyte donor's *FMR1* status to prevent transmission of maternal premutation range (CGG_n∼55–200_) and/or expansions to full mutations (CGG_n>200_; fragile X syndrome; FXS) and other associated neuro-psychiatric complications, mostly affecting males [1]. Such testing is, however, usually only performed after a donor has already been selected. Here we suggest that, if further studies confirm here reported data, *FMR1* testing should be performed in oocyte donor candidates as a tool of primary selection.

Finally, here presented differences in distribution of *FMR1* mutations also further demonstrate the negative impact of *low* alleles on the *FMR1* gene on female fertility. While middle aged infertility patients in section (iii) of this study and presumed fertile women in section (ii) present with quite similar genotype/sub-genotype distribution, older infertile women in section (i), with mean age 39.7±5.7 years, demonstrated approximately 20 percentage points lower *norm FMR1* genotype prevalence (41.1%) and a much higher prevalence in *het-norm/low* sub-genotype (30.1%. equaling the prevalence of *het-low* and *het-high* sub-genotypes combined in the younger group of infertile women).

These data, therefore, suggest that, as infertile women age, those who remain in treatment are increasingly adversely selected: those with *norm* genotypes and best pregnancy chances decline in prevalence, and women with *low* alleles, and poorer pregnancy chances, increase in prevalence. As noted before, this patient distribution is also likely reflective of our center's highly adversely selected patient population. Older reproductive age women with *low FMR1* alleles, who disproportionally fail to conceive, can be expected to “accumulate” in a center like ours, which generally is considered a center of “last resort” for patients who have previously failed elsewhere. A younger patient population, demonstrated in section (ii) reflects women with lower dropout rates and, therefore, fewer *low FMR1* alleles and more norm genotypes. This is consistent with our recent finding that young infertile women with *low* alleles disproportionately dropout from infertility treatment [6].

We in this discussion outlined the principal weaknesses of this study. Likely the most important being the small size of some patient subgroups in the three investigated patient populations. The statistical robustness of here reported findings is, therefore, that more remarkable. Our data, nevertheless, require confirmation. They, however, suggest an increasingly important impact of the *FMR1* gene on ovarian aging and female fertility and infertility.
